# Absorption of the Liquor Amnii—Descent of the Gravid Uterus

**Published:** 1835

**Authors:** 


*E Sylvis Nuncius.*

---

CINCINNATI, OCTOBER 1, 1835.

---

### ART. I.—ABSORPTION OF THE LIQUOR AMNII—DESCENT OF THE GRAVID UTERUS.

EVERY obstetrician must have met with cases in which, at the time of parturition, there was little or no discharge of liquor amnii. An instance of this kind lately occurred in our own practice. The patient nearly 40 years old, not before in a state of gestation, was so large at the beginning of the ninth month, as to excite the expectation of twins. Two or three weeks before delivery, she began to diminish in size, and in a few days was reduced to a degree that struck her as remarkable. As the labor advanced, the aqueous tumor in front of the head of the fœtus, was barely sufficient to show the integrity of the membranes, and when we ruptured them scarcely an ounce of fluid escaped; there was no perceptible discharge during the subsequent pains, and none followed the escape of the fœtus.

The absorption of the liquor amnii, to a greater or less extent, in the latter days of gestation, is no doubt a constant physiological fact, of which our obstetrical writers seem to have taken very little notice. The diminution of size in the uterine tumor is generally ascribed to the subsidence into the pelvis of the head of the fœtus; but such a sinking down, if in fact it occurs at all, must be too little to explain the great reduction of size in the abdomen:—that reduction we are convinced is chiefly the effect of absorption. As the remaining waters would of course be accumulated in the lower part of the uterus, the size of the patient, measured round the ossa ilia, would, except in the few cases in which the absorption was perfect, be nearly the same as before, while her circumference between the umbilicus and epigastrium would be lessened, thus giving the appearance of subsidence into the pelvis.

The objects of nature in this absorption seem to be threefold. First, by taking off the tension of the uterus that organ can accommodate its figure to the descent of the head, into the superior straight of the pelvis, which it could not do when distended:—Second, the diminution favors uterine contraction, in the same manner as removing, with the catheter, a portion of urine, in ischuria, enables the bladder to contract on the remainder, and empty itself. The immediate effect of the escape of a portion of the liquor amnii during labor, in augmenting the expulsive power of the uterus, is familiar to all practitioners, and depends on the same cause. By the way, we feel inclined, as the opportunity here offers, to take exception to the rule, copied from one system of obstetrics into another, that the membranes are not to be ruptured till very near the close of labor. Whenever the uterine tumor is very great, and the expulsive pains inefficient, a comparatively early rupture of the membranes facilitates the labor, by enabling the organ to contract with greater power. But let us return to the third and last end of nature, in the absorption of the liquor amnii. This is the greater safety of the mother, whose, exhaustion, immediately after delivery, will, *cæteris paribus*, be indirectly as the amount which then suddenly escapes from the uterus.

